# Temperature and Preparation Time Intervals on Survival of *Opisthorchis viverrini* Metacercariae in Pickled Fish (Pla-som)

**DOI:** 10.1155/2024/4817012

**Published:** 2024-11-12

**Authors:** Naiyana Senasri, Nattiya Chumnanka, Wiboonsuk Talkul

**Affiliations:** ^1^Department of Fisheries, Faculty of Natural Resources, Rajamangala University of Technology Isan Sakon Nakhon Campus, Sakon Nakhon 47160, Thailand; ^2^Health Science Program, Faculty of Science and Technology, Sakon Nakhon Rajabhat University, Sakon Nakhon 47000, Thailand

## Abstract

This study involved two series of independent trials that investigated the storage temperature and time of raw fish to eliminate *Opisthorchis viverrini* metacercarial infection in Thai pickled fish. A total of 330 healthy silver barb fishes (30-day posthatch) were infected with *O. viverrini* cercariae at an inoculation ratio of 50 cercariae/fish. After infection, the fish was reared for 3 months. The first trial was to evaluate the effect of fish storage temperature in the recovery from *O. viverrini* metacercariae. The infected fishes were randomly divided into five groups and stored at −20°C, 2°C, 4°C, and 8°C or room temperature (as a control) for 24 h. The results showed that at a storage temperature of −20°C, the fish initially had a reduced recovery rate from *O. viverrini* metacercariae. The second trial investigated the storage time for the recovery of infected fish stored at −20°C for 18, 24, 36, 48, or 60 h and used room temperature as a control group. The results revealed that storing infected fish at −20°C for 48 and 60 h had the lowest recovery rate from *O. viverrini* metacercariae at 0.00%. In conclusion, storing infected fish at a temperature of −20°C for a period of 48 h or longer could restrict the *O. viverrini* metacercaria recovery rate. These results were recommended as suitable conditions for the preparation of Pla-som to avoid *O. viverrini* metacercaria contamination.

## 1. Introduction

The zoonotic fish-born trematode *Opisthorchis viverrini* is an emerging parasite that develops into liver flukes in humans. The *O. viverrini* infection is distributed in Lower Mekong subregion countries, particularly Thailand, Laos, Cambodia, and Vietnam, which have reported over 10 million cases of infection in humans [1]. The most severe complication of chronic and acute *O. viverrini* infection could develop bile duct cancer or cholangiocarcinoma (CCA) [2, 3]. Northern and northeastern regions of Thailand have the highest reports of *O. viverrini* infection rates, with 6–8 million cases [3–5]. Liver fluke is particularly prevalent for the local people of the northeastern region of Thailand. The public health campaigns from across the Thai governments and nongovernmental organizations have revealed the risk of liver fluke in order to control *O. viverrini* infection. The prevalence of infection has not decreased [2, 3] because it might be challenging to alter culturally ingrained eating habits. The consuming behavior of raw fish, contaminated with *O. viverrini* metacercaria infective stage, has been associated with liver fluke infection [6]. After ingestion of food contaminated with *O. viverrini* metacercaria, which is infective for humans, the fully adult fluke in biliary ducts deposits embryonated eggs that pass through the feces and are released to the environment. Embryonated eggs release miracidium when they are ingested by snail *Bithynia* sp. the first intermediate host of *O. viverrini*, distributed in northeast of Thailand [7, 8], and develop into sporocysts, rediae, and cercariae, respectively. Cercariae, released from the first intermediate host, encyst as metacercariae in the muscles after penetrating the second intermediate host, especially cyprinoid fish. Metacercaria is the infective stage for humans who eat raw or undercooked fish. After infection, it develops into the adult stage in the human small bile ducts [1].

Many fish species in the family Cyprinidae serve as second intermediate hosts for *O. viverrini* with various intensity and prevalence of infection [4, 9]. People in the north and northeast of Thailand preserve freshwater fishes by fermentation with salt as the main ingredient such as Pla-ra and Pla-som [10] which are sources of infection. Those fishes of Cyprinidae are also made as fermented fishes and are consumed as raw or semicooked with steamed sticky rice [11, 12]. For the preparation of Pla-som, they marinated cyprinid fish with salt and sticky rice for 3 days or more. The fish develops a distinct sour taste as a result of increased acid levels during preservation [13]. However, liver fluke infections are mostly found in cyprinoid fish, which is the desired species for making Thai pickled fish (Pla-som). Pla-som is usually fermented within 1 week before eating, and the duration of fermentation might affect the ability of the infection of the metacercariae. This indicates that Pla-som is at a high risk of infection with liver fluke metacercariae [13]. Thus, to avoid carcinogenic liver fluke metacercaria contamination in food, the present study is aimed at investigating the effect of storage temperature to diminish the infection in Thai pickled fish.

## 2. Materials and Methods

### 2.1. Ethic Approval

The study protocols and design were approved by the Institutional Animal Care and Use Committee of Rajamangala University of Technology Isan, based on the Ethics of Animal Experimentation of National Council Research of Thailand (Approval ID Proposal 14/2564).

### 2.2. Effect of Various Storage Temperatures on Survival of Metacercariae in Pla-som

#### 2.2.1. Fish and *O. viverrini* Cercaria Infection

**Figure 1:**
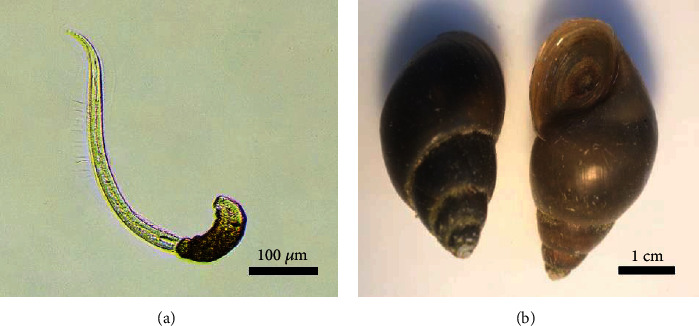
(a) *Opisthorchis viverrini* cercaria (scale bar = 100 *μ*m) by cercarial shedding. (b) *Bithynia siamensis goniomphalos* (scale bar = 1 cm) from the irrigated area collection.

The hatched larvae of Silver barb (*Barbonymus gonionotus*) were obtained from in-house hatchery at Rajamangala University of Technology Isan, Sakon Nakhon Campus. The larvae were grown in 5 t of circular concrete ponds until 30 days of age [14]. Five hundred *O. viverrini*–free fish larvae (size 4–5 cm.) were infected with *O. viverrini* cercaria (Figure 1(a)). The infection ratio was one fish/50 *O. viverrini* cercariae for 24 h of exposure [14, 15]. The *O. viverrini* cercariae were collected from freshwater snail *Bithynia siamensis goniomphalos* by cercarial shedding (induced by exposure to electric light (40 W) for 2–3 h) and counted under a stereomicroscope for use in fish infection (Figure 1(b)). We have identified it under a light microscope using the morphological characteristics of a tobacco pipe shaped when briefly hanged head down or laid on the bottom, a pair of eyespots, and a long tail with fins on both lateral sides. [8, 16, 17]. After *O. viverrini* infection, all fishes were transferred to a 5-t fiber pond and reared for 3 months (size 12–15 cm.).

#### 2.2.2. Assessment of *O. viverrini* Metacercaria Recovery Under Different Temperatures

After 3 months, the 150 infected fishes were randomly distributed into five groups (three replicates/group, 10 fish each replicate). Cold shock method, using ice-shocked water (0°C), was used for euthanizing fish prior to storage in various temperatures. Store at different temperatures of 2°C, 4°C, 8°C, or −20°C for 24 h in a temperature-controlled cabinet, using room temperature (25°C) as a control group. After the storage was under different temperatures, four treatments of the *O. viverrini*–infected fish were collected in a tube, grinded, and digested in pepsin solution (0.25%pepsin + 0.85%NaCl + 1.5%HCl). The samples were mixed and incubated in shaking water bath at 37°C for 1 h until homogeneous. The digested samples were filtered with 1000, 300, 106, and 250 *μ*m of sieve. The sieved sediments were then cleaned with NaCl 0.85% until clear. Ultimately, the metacercariae in the sediment were isolated and identified under a stereomicroscope [18]. The isolated recovered *O. viverrini* metacercariae were collected, stained with trypan blue, and observed using a stereomicroscope.

### 2.3. Study of Impact of Storage Time on Metacercaria Recovery for Production of Thai Pickle Fish

#### 2.3.1. Experimental Conditions and Assessment of *O. viverrini* Metacercaria Recovery Storage Time

Five hundred fish larvae were infected with *O. viverrini* cercariae. One hundred and eighty fish with *O. viverrini* infection with iced water (0°C) prior to use in the experiment. Ten fishes with *O. viverrini* infections were randomly divided into six experimental groups, including a control, and run in triplicate. The experimental was designed with increasing period of time for storage of *O. viverrini–*infected fish at 18, 24, 36, 48, and 60 h. After the storage time, six treatments of the *O. viverrini*–infected fish methods were followed by Section 2.2.2.

#### 2.3.2. Morphology of Metacercariae

An *O. viverrini* metacercaria from sediment was collected to study its morphology including cyst dimension, the active move of encysted metacercariae, oral sucker, ventral sucker, and scattered brown pigments [19]. The number of *O. viverrini* metacercariae of each treatment was collected for comparison of *O. viverrini* metacercaria infectivity, infection intensity, and recovery rate.

### 2.4. Data Analysis

The data were collected to analyze the differences in infection rates of liver fluke in experimental groups by finding the percentage of liver fluke infection, the intensity of liver fluke infection (metacercaria per fish), and the percentage of recovery of liver fluke at the metacercaria.

 $$\begin{array}{c} \text{Infection rates}=\text{number of infected fish}\times 100/\text{total examined fish} \end{array}$$

 $$\begin{array}{c} \text{Intensity of the infection}=\text{total metacercarial}/\text{total infected fish} \end{array}$$

 $$\begin{array}{c} \text{Percentage of metacercarial recovery}=\text{total metacercarial recovery}\times 100/\text{total cercarial} \end{array}$$

### 2.5. Statistical Analysis

Data of the intensity, percentage of metacercaria infection, and recovery rate from metacercariae in fish were presented in mean ± SD. Statistical analysis was analyzed by one-way ANOVA using SPSS 11.0 for Windows (SPSS Inc., Chicago, IL, United States). A *p*-value less than 0.05 was considered statistically significant.

## 3. Results

### 3.1. The Effect of Various Storage Temperatures of Fish Tissue on Metacercaria Recovery

The result of five storage temperatures of fish tissue on metacercaria recovery for 24 h is shown in Table 1. The storage temperature of *O. viverrini*–infected fish at −20°C, 2°C, 4°C, and 8°C showed the infectivity increased from 90.00 ± 0.00% to 93.33 ± 5.77%, where *O. viverrini*–infected fish storage at room temperature showed the highest infectivity at 96.67 ± 5.77%. Among all groups, the highest infection intensity was observed in the fish stored at room temperature (control group) at 13.10 ± 1.73 metacercariae/fish. The lowest infection intensity was observed in the fish stored at 4°C at 9.74 ± 0.73 metacercariae/fish. The intensity of infection in fish was significantly different (*p* < 0.05), except for the fish stored at 8°C that was not statistically different. Moreover, the percent recovery of metacercariae in the control group was significantly different (*p* < 0.05) in the fish stored at −20°C, 2°C, 4°C, and 8°C. The maximum rate of *O. viverrini* metacercaria recovery showed in the control group at 25.33 ± 1.70%, followed by storage temperature of *O. viverrini*–infected fish at 8°C, 4°C, and 2°C which were 17.67 ± 1.33%, 17.53 ± 0.11%, and 14.47 ± 0.11%, respectively. The storage temperature of *O. viverrini*–infected fish at −20°C presented the lowest recovery rate of metacercariae at 2.93 ± 0.64% (Table 1).

**Figure 2:**
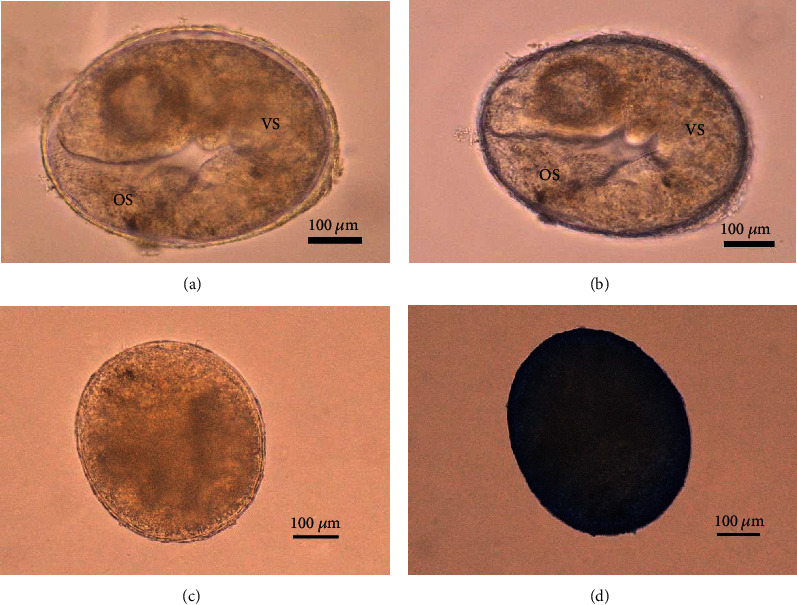
Staining *O. viverrini* metacercariae using trypan blue. (a, b) Room temperature (control group), (a) prestained (normal) and (b) stained. (c, d) Storage temperature at −20°C, (c) prestained and (d) stained. OS = oral sucker, VS = ventral sucker. Scale bar is 100 *μ*m.

Moreover, the oral sucker and ventral sucker could be distinguished in the *O. viverrini* metacercariae collected from infected fish stored at room temperature (control group) and 8°C, 4°C, and 2°C as observed under a stereoscope. However, the *O. viverrini* metacercariae collected from infected fish stored at −20°C were found to have ruptured encysted cells. The *O. viverrini* metacercariae were confirmed dead by staining with trypan blue. The *O. viverrini* metacercariae of the control group showed an unstained cell, while the *O. viverrini* metacercariae collected from infected fish stored at −20°C was considered dead from the stained cell by trypan blue (Figures 2(a), 2(b), 2(c), and 2(d)).

### 3.2. Study of Impact of Storage Time on Metacercaria Recovery for Production of Thai Pickle Fish

The storage temperature of −20°C showed the lowest recovery rate of *O. viverrini* metacercariae for production of Thai pickled fish (results of the study of effect of various storage temperatures of fish tissue on metacercaria recovery). It was thereby used as the suitable storage temperature to study the impact of storage time on metacercaria recovery for producing Thai pickled fish. The results showed that most of the infectivity of *O. viverrini* metacercariae occurred in the control group at 90.00 ± 0.00%. It was followed by the *O. viverrini* metacercaria–infected fish stored for 18 h with 83.3 ± 11.5% infectivity. The *O. viverrini* metacercaria–infected fish stored for 24, 36, 60, and 48 h showed slight decreased infectivity at 80.00 ± 10.00%, 80.00 ± 0.00%, 76.67 ± 5.77%, and 73.33 ± 5.77%, respectively, which were significantly different (*p* < 0.05) (Table 2). In addition, the infection intensity was found to be significantly different (*p* < 0.05). The result of infection intensity was similar to that of infectivity, the *O. viverrini* metacercaria–infected fish of the control group had the highest infection intensity at 12.18 ± 0.39 metacercaria/fish. It was followed by the *O. viverrini* metacercaria–infected fish stored for 18, 24, 60, 36, and 48 h with 12.13 ± 0.21, 11.93 ± 0.11, 10.70 ± 0.23, 10.68 ± 0.28, and 10.67 ± 0.51 metacercaria/fish, respectively (Table 2). The recovery rate of *O. viverrini*–infected fish in the control group and the group stored for 18 h was the highest at 21.93 ± 0.70% and 20.20 ± 2.61%, respectively, which were significantly different (*p* < 0.05) compared to the groups stored for 24, 36, 48, and 60 h which were 3.27 ± 2.04%, 3.20 ± 3.12%, 0.00 ± 0.00%, and 0.00 ± 0.00%, respectively. In this study, the recovery rate of *O. viverrini* metacercariae was impacted by storage time of 48 and 60 h which indicated the 0.00 ± 0.00% of *O. viverrini* metacercaria recovery (Table 2).

**Figure 3:**
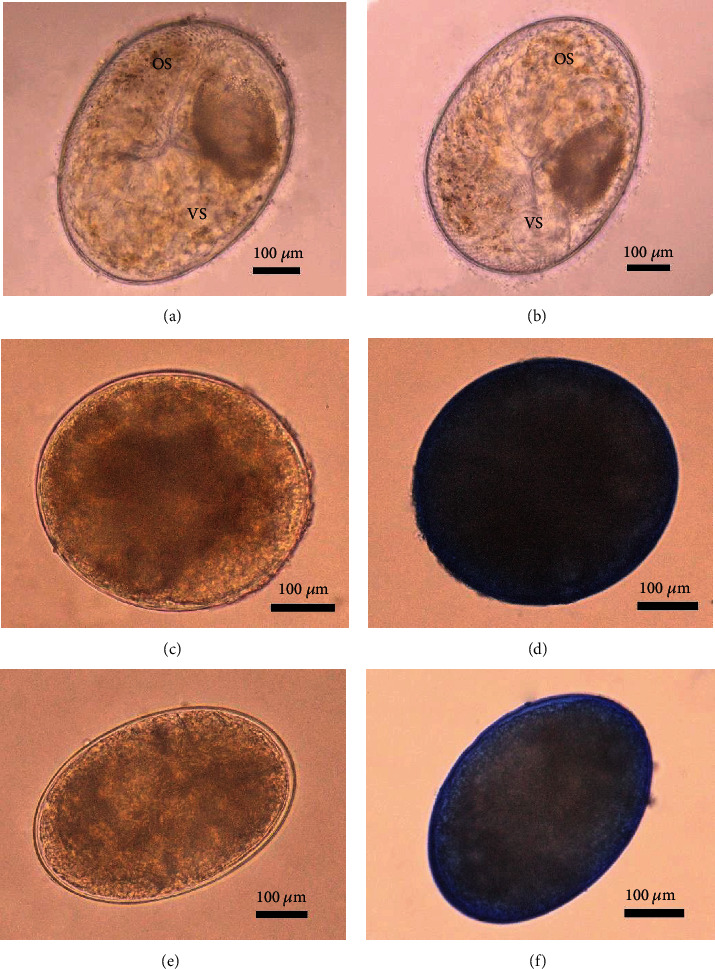
Staining of *O. viverrini* metacercariae collected from different storage times of *O. viverrini* metacercaria–infected fish at −20°C using trypan blue. (a, b) Room temperature (control group), (a) prestained (normal) and (b) stained. (c, d) Storage time for 48 h, (c) prestained and (d) stained. (e, f) Storage time for 60 h, (e) prestained and (f) stained. OS = oral sucker, VS = ventral sucker. Scale bar is 100 *μ*m.

The oral sucker and ventral sucker *O. viverrini* metacercaria cysts collected from infected fish in the control group could be distinguished. On the other hand, the infected fish in other groups stored at −20°C for 48 and 60 h did not show recovery and the cysts were ruptured. The cysts were stained with trypan blue to confirm cell death of *O. viverrini* metacercariae. The *O. viverrini* metacercariae in the control group were not stained blue (Figure 3). The *O. viverrini* metacercaria cyst from the infected fish stored for 48 and 60 h was ruptured and could be stained blue (Figure 3).

## 4. Discussion

In this present study, *O. viverrini* metacercaria–infected fish were stored at different times and temperatures and their effects on *O. viverrini* metacercaria infectivity, intensity of the infection, and metacercaria recovery investigated. The *O. viverrini*–infected fish stored at −20°C for 24 h gave the lowest recovery rate. This was similar to the study by Onsurathum et al. [13], on the effect of treating fish in low temperature on the morphology and infectivity of *O. viverrini* metacercariae. The infectivity of *O. viverrini* metacercariae might be affected by pretreating with low temperature, as reported in several trematodes [20, 21]. The highest infection intensity of *O. viverrini* metacercariae (13.10 ± 1.73 metacercariae/fish) was found in the infected control group and could be observed in several organs in the fish body. A similar report on the high intensity of infection of *O. viverrini* metacercariae was published by Tesana et al. [22].

The infectivity and recovery rate of *O. viverrini* metacercariae could be governed by preparation time and cooking procedures. From this study, it was found that fishes were stored at −20°C for 18, 24, and 36 h; still recovered; and were able to be infected (20.20 ± 2.61%, 3.27 ± 2.04%, and 3.20 ± 3.12%, respectively). Viable *O. viverrini* metacercariae were examined by staining with trypan blue. The cell with degradation could be stained blue, whereas viable metacercariae could not be stained by trypan blue [23, 24]. The results in this study were similar to those in the study of Onsurathum et al. [13], which investigated the storage of *O. viverrini* metacercaria fish. The results from the study indicated that the viable *O. viverrini* metacercaria–infected fish stored for 3 days retained the host-invasive function and could infect a hamster. The structure of *O. viverrini* metacercariae in the group of infected fish stored for 3 to 5 days was completely destroyed and could not infect the host. In this study, the recovery rate of *O. viverrini* metacercariae was markedly inhibited storing the infected fish at −20°C for 48 h. Long storage duration of the fish in low or freezing temperature causes the *O. viverrini* metacercariae to lose their ability to harm. Low or freezing temperatures could transform the liquid within the cell to solid hence destroying the cyst structure [25]. To confirm that the metacercariae were damaged, the cyst was stained with blue color [23] and the organs became undistinguishable. Similarly, according to Onsurathum et al. [13], a longer storage period of 6–7 days of fermentation process not only increased the percentage of metacercaria degradation but also decreased the recovery rate of metacercariae in the first 3–7 days of fermentation process. The metacercariae were not found in Pla-som (salt fermented) after 3, 7, and 14 days of fermentation [12]. An increase in total acidity and viability effect and infectivity of *O. viverrini* metacercariae during the production and fermentation could not effectively eliminate the death of metacercariae [26]. On the contrary, the traditional fermented cyprinoid fish with salt for 48 h could be effective in hindering metacercariae of intestinal fluke *Haplorchis taichui* [27].

In conclusion, the traditional eating of Thai pickled fish (Pla-som) carries a high risk of *O. viverrini* metacercaria infection by food contamination but storing of fish at −20°C could reduce the infectivity of *O. viverrini* metacercariae, which is the cause of a carcinogenic liver fluke.

## Figures and Tables

**Table 1:** The storage temperature of *O. viverrini* metacercaria–infected fish on metacercaria infectivity (*n* = 30).

**Temperature (°C)**	**Infectivity of *O. viverrini* metacercariae (%)**	**Number of infection intensity (metacercariae/fish)**	**Recovery rate of *O. viverrini* metacercariae (%)**
Control	96.67 ± 5.77	13.10 ± 1.73^a^	25.33 ± 1.70^a^
−20	93.33 ± 5.77	11.21 ± 0.01^b^	2.93 ± 0.64^d^
2	93.33 ± 5.77	10.72 ± 0.06^b^	14.47 ± 0.11^c^
4	90.00 ± 0.00	9.74 ± 0.73^c^	17.53 ± 0.11^b^
8	90.00 ± 0.00	9.81 ± 0.30^c^	17.67 ± 1.33^b^

*Note:* Values in the same vertical line with different superscripts differ significantly (*p* < 0.05).

**Table 2:** The impact of storage time of *O. viverrini* metacercaria–infected fish on metacercaria infectivity (*n* = 30 fishes).

**Storage time (h)**	**Infectivity of *O. viverrini* metacercariae (%)**	**Number of infection intensity (metacercaria/fish)**	**Recovery rate of *O. viverrine* metacercariae (%)**
Control	90.00 ± 0.00^a^	12.18 ± 0.39^a^	21.93 ± 0.70^a^
18	83.33 ± 11.54^ab^	12.13 ± 0.21^a^	20.20 ± 2.61^a^
24	80.00 ± 10.00^ab^	11.93 ± 0.11^a^	3.27 ± 2.04^b^
36	80.00 ± 0.00^ab^	10.68 ± 0.28^b^	3.20 ± 3.12^b^
48	73.33 ± 5.77^b^	10.67 ± 0.51^b^	0.00 ± 0.00^b^
60	76.67 ± 5.77^ab^	10.70 ± 0.23^b^	0.00 ± 0.00^b^

*Note:* The values in the same vertical line with different superscripts differ significantly (*p* < 0.05).

## Data Availability

Data sharing is not applicable to this article as no datasets were generated or analyzed during the current study.
